# 3D Bioprinting: An Enabling Technology to Understand Melanoma

**DOI:** 10.3390/cancers14143535

**Published:** 2022-07-20

**Authors:** Samantha Fernandes, Cian Vyas, Peggy Lim, Rúben F. Pereira, Amaya Virós, Paulo Bártolo

**Affiliations:** 1 Department of Mechanical, Aerospace and Civil Engineering, University of Manchester, Oxford Road, Manchester M13 9PL, UK; samantha.fernandes@manchester.ac.uk (S.F.); cian.vyas@manchester.ac.uk (C.V.); peggylim317@gmail.com (P.L.); 2 Singapore Centre for 3D Printing, School of Mechanical and Aerospace Engineering, Nanyang Technological University, Singapore 639798, Singapore; 3 ICBAS—Instituto de Ciências Biomédicas Abel Salazar, Universidade do Porto, 4050-313 Porto, Portugal; ruben.pereira@ineb.up.pt; 4 i3S—Instituto de Investigação e Inovação em Saúde, Universidade do Porto, 4200-135 Porto, Portugal; 5 INEB—Instituto de Engenharia Biomédica, Universidade do Porto, 4200-135 Porto, Portugal; 6 Skin Cancer and Ageing Laboratory, Cancer Research UK Manchester Institute, University of Manchester, Oxford Road, Manchester M13 9PL, UK; amaya.viros@cruk.manchester.ac.uk

**Keywords:** 3D printing, bioprinting, disease models, melanoma, skin equivalents

## Abstract

**Simple Summary:**

Melanoma is a form of skin cancer that has increased in incidence in the last few decades. The main environmental risk factor is exposure to ultraviolet radiation (UVR). It is the fifth most common cancer in the UK, with a 17% mortality rate. There are new melanoma therapies that show improvement in patient survival; however, there is a significant proportion of patients who do not respond to approved treatments, for whom there are no second line therapies. Developing safe new therapies without significant side effects for patients is a pressing clinical challenge; 3D skin equivalents allow for disease modelling and systematic and safe drug testing for skin cancer. This paper reviews recent advances in creating 3D skin and cancer models for effective drug screening for melanoma.

**Abstract:**

Melanoma is a potentially fatal cancer with rising incidence over the last 50 years, associated with enhanced sun exposure and ultraviolet radiation. Its incidence is highest in people of European descent and the ageing population. There are multiple clinical and epidemiological variables affecting melanoma incidence and mortality, such as sex, ethnicity, UV exposure, anatomic site, and age. Although survival has improved in recent years due to advances in targeted and immunotherapies, new understanding of melanoma biology and disease progression is vital to improving clinical outcomes. Efforts to develop three-dimensional human skin equivalent models using biofabrication techniques, such as bioprinting, promise to deliver a better understanding of the complexity of melanoma and associated risk factors. These 3D skin models can be used as a platform for patient specific models and testing therapeutics.

## 1. Introduction

The human skin is the outermost covering of the body and the largest organ of the integumentary system, protecting underlying tissues and organs [1]. As a barrier to the environment, skin plays a vital role in protecting the body against external pathogens and ultraviolet radiation (UVR). However, skin is prone to infections and disorders, including skin cancers [2].

Among the different skin cancers that affect the human body, melanoma is one of the most aggressive and has a poor prognosis when it invades the dermis [2]. The primary environmental driver of cutaneous melanoma arising over hair-bearing skin is exposure to UVR (direct sun exposure or through devices, such as tanning beds) in susceptible populations, which are predominantly fair skinned people of European descent [3,4]. The specific link between environmental triggers and non-cutaneous melanoma subtypes, which arise over completely sun protected or sun shaded sites, is unknown. Melanoma is one of the fastest growing cancers worldwide with an overall increase of 3–6% in the last few decades [5]. In 2019, melanoma was the 19th most commonly occurring cancer worldwide in both men and women [6]. It is characterised by high complexity and heterogeneity as there are several mutations in genes and activation of signalling pathways that cause tumour progression which is a therapeutic challenge [7,8]. Further complexity arises from the cellular interactions between the tumour and its surrounding microenvironment [9]. The tumour microenvironment (TME) consists of various types of non-cancer cells and their stroma, such as extracellular matrix (ECM), fibroblasts, immune cells, mesenchymal cells, and endothelial cells. Such cell–cell and cell–ECM interactions are complex and dynamic in nature, resulting in spatiotemporal alterations of the TME at the physiological, structural, and functional levels [9]. The tumour, along with its microenvironment, induce and sustain the processes of tumour development and progression [10]. Therefore, it is important to understand the interactions between the tumour and the TME on a three-dimensional (3D) level so that complex features, such as cell migration, proliferation, and chemoresistance can be investigated in conditions that closely recapitulate the native melanoma TME [10].

This paper aims to provide an overview of the role of bioprinting technologies to fabricate biomimetic 3D models of melanoma to enable advances in understanding melanoma, screening of new drugs and precision medicine. First, we highlight the mechanisms underlying malignant melanoma onset and progression, providing the fundamental knowledge to understand the heterogeneity and complexity of the melanoma tumour microenvironment. Subsequently, the most used approaches for in vitro melanoma modelling are reviewed regarding their ability in reproducing key features of melanoma. Lastly, recent advances in bioprinting of melanoma models are discussed, along with the challenges and future opportunities in the field.

## 2. Melanoma

Malignant melanoma arises from melanocytes, which are pigment-producing cells that are primarily located in the epidermis, but can also be found in the dermis, and occasionally at rare visceral sites [11]. Thus, it typically affects the skin, the uvea and retinal pigmented epithelium, mucosae and very rarely visceral organs [12,13,14,15].

The cutaneous subtype is the most frequent clinical subtype in the UK, mainly affecting individuals of European descent, and once it metastasises, the prognosis is poor [8]. Therefore, early diagnosis and early therapies are crucial to outcome [8]. Sun exposure, mainly during childhood and adolescence, can induce melanocytic naevi (benign melanocytic tumours) in individuals who are genetically susceptible, and increased numbers of naevi are associated with a higher risk of melanoma [16]. According to a meta-analysis conducted by Gandini et al. [17], individuals with more than 100 melanocytic naevi have a sevenfold risk for lifelong melanoma development.

Cutaneous melanomas can be broadly categorised by their origins on the hair-bearing skin that has been chronically sun-damaged (CSD) or only intermittently sun exposed (non-chronically sun-damaged skin; non-CSD) [18]. There are clinical and epidemiological hallmarks that arise associated with these two main categories of cutaneous melanoma [19]. The main genetic drivers for cutaneous melanoma are B-Raf proto-oncogene (BRAF), neurofibromin 1 (NF1), and neuroblastoma RAS viral oncogene homolog (NRAS) mutations, which are primarily mutually exclusive in primary tumours [20]. CSD melanomas tend to arise in more elderly patients, with a history of multiple other non-melanoma skin cancers, a long history of accumulated sun exposure, and are more frequently driven by either gain of function NRAS mutations, loss of function NF1 mutations or BRAF gain of function mutations. Melanomas associated with CSD skin have a high mutational load, which is related to accumulated, chronic UVR exposure, and generally occur on sun exposed areas, such as the head, neck, and upper dorsal region [18,20]. In contrast, non-CSD melanomas arise in younger individuals (age < 55) in intermittently sun exposed areas of the body, such as the trunk and proximal extremities; and arise more frequently in very fair individuals harbouring germline variants in *MC1R* (the red hair gene) [18]. Non-CSD melanomas are usually associated with *BRAF^V600E^* and exhibit a comparatively lower mutational load [19]. The progression of melanocytic neoplasia, from normal melanocytes to metastatic melanoma is a multistep process that involves alterations, and additive genomic changes in multiple cancer genes (Table 1) [21].

### 2.1. Melanocytes and Melanin

**Figure 1:**
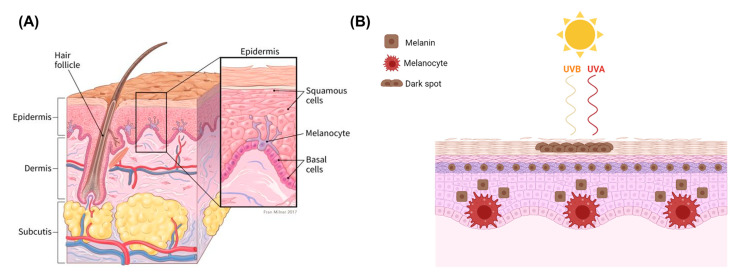
(**A**) Structural anatomy of human skin showing the three main layers: epidermis, dermis, and hypodermis. Structure of the epidermis showing melanocytes residing between squamous and basal cells. Melanocytes are responsible for the synthesis of melanin in pigment granules called melanosomes, which are transported to keratinocytes for protection from UVR (American cancer society, 2021). (**B**) Schematic representation of melanin production due to UVA (315–400 nm) and UVB (280–315 nm). UVA penetrates deeper into the skin, playing a key role in premature skin ageing (photoageing) contributing also to the development of skin cancer. UVB is responsible for sunburns and a major cause of skin cancer, particularly malignant melanoma (image created using Biorender).

Melanocytes derive from the neural crest during early embryonic development and primarily reside in the basal layer of the epidermis (Figure 1A), middle layer of the eye, inner ear, meninges, bones, and heart [31,32,33]. The major function of melanocytes is the production of melanin. In humans, melanin is contained in melanosomes, which are transferred to the surrounding keratinocytes. When UVR inflicts DNA damage to keratinocytes and p53 is upregulated, keratinocytes secrete α-melanocyte stimulating hormone (α-MSH) [34,35].

α-MSH is secreted from keratinocytes and binds to the melanocortin 1 receptor (MC1R) in melanocytes, which is a G-protein coupled receptor that induces the synthesis of melanin in response to UVR damage initially detected by keratinocytes (Figure 1B) [36,37,38]. In parallel to switching on eumelanin (dark melanin) production and driving the tanning response, activation of *MC1R* also activates DNA damage repair [36,39,40,41]. Recent work has shown that individuals carrying germline loss-of-function variants in *MC1R* have reduced signalling and synthesise more phaeomelanin, or light, red melanin. The higher ratio of phaeomelanin to eumelanin in *MC1R* carriers leads to fairness in complexion, hair, and eyes, as well as photosensitivity. Individuals who are *MC1R* carriers are at higher risk of melanoma [36,42,43,44].

Eumelanin is an effective absorbent of light that can disperse over 99.9% of UVR [34]. Therefore, epidermal keratinocytes use melanin, transferred from melanocytes, to protect their nucleus from UVR-induced DNA damage and mutations [34]. UVR causes DNA mutations that accumulate over time, and therefore, it is a paramount risk factor for malignant melanoma and other skin cancers [6]. UVB and UVA radiation penetrate the epidermis and dermis of darker skin less efficiently, with UVB decreasing by approximately 50% in darker skinned epidermis, and UVA decreasing from 27% to 4% at wavelengths of 314 nm and from 47% to 14% at 400 nm wavelengths [45]. A lack of protection from UVR causes damage to connective tissue and DNA underneath the skin which can contribute to premature ageing as well as skin cancer [46,47,48].

### 2.2. Mechanisms of Melanoma Progression and Physiology

Melanoma has a high mutational burden due to the UVR induced DNA damage and DNA replication errors [49]. These mutations contribute to different features of melanocytic neoplasia. Intriguingly, the key driver mutations initiating tumourigenesis (*NRAS, BRAF*) are not driven by UVR damage and the trigger of these is unknown [49].

Melanocytes with mutations can sometimes progress to form benign melanocytic tumours called naevi (Table 2). Most naevi are harmless and do not progress to melanoma. Additional mutations, however, primarily due to repeated sun exposure, can transform naevi to melanoma in situ, which are malignant but localised melanocytic tumours that have not yet crossed the basement membrane. Melanomas in the epidermis frequently have a period of radial growth along the epidermis, but if sufficient genetic hallmarks of disease accumulate, the tumours acquire the capacity to enter the dermis and then spread either via the lymphatics or via direct haematogenous spread [18,50,51]. This course of disease progression, however, does not encompass all possible pathways to melanoma. A large proportion of primary cutaneous melanomas do not arise over previously existing naevi and are termed “de novo” melanomas [52].

Skin ageing is a biological process influenced by several intrinsic and extrinsic factors over time [54,55]. It is estimated that the epidermal thickness is reduced by ~6.4% per decade, which in turn causes shorter and flatter keratinocytes and larger corneocytes. Melanocyte activity is also decreased by ~8–20% per decade [54]. In the dermis, the synthesis of collagen and elastin decreases with age, causing aged skin to be less elastic.

Cancer is often defined as a disease of ageing, as the probability of developing invasive cancer in individuals over 60 years of age is over double that of patients who are younger than 60 [56]. Tumourigenesis is accelerated by the build-up of genetic and epigenetic damage, and similarly, ageing is associated with accumulated damage to intracellular large molecules. Macromolecular damage initially affects lipids, DNA and cellular proteins, and also impairs the tissue regeneration process [57].

One of the hypotheses that explains the link between ageing and cancer is that ageing likely produces a more favourable microenvironment that enables cancer progression and aggressiveness since both processes share a similar mechanism of time dependent accumulation of cellular damage. To test this hypothesis, Gomes et al. [58] cultured human cancer cell types A549 and HCC1806 in 10% human serum from either young (age ≤ 30) or older donors (age ≥ 30). Results showed that 83% of cells which were treated with young donor serum maintained their morphology, whereas 83% of the cells treated with old donor serum transitioned into mesenchymal cells. Therefore, cells exposed to aged serum displayed the epithelial to mesenchymal transition (EMT) [58]. This suggests that the root cause of both ageing, and cancer is the accumulation of cellular damage.

Although cancer and ageing share common mechanisms, establishing a direct causal link is challenging. It is possible that the ‘convergent’, or shared molecular hallmarks, such as the accumulation of cellular damage that has occurred over time, will drive both the ageing and the cancer phenotypes. However, there are other ‘divergent’ or unique mechanisms that have an opposing influence on cancer and ageing; especially protecting the individual from cancer but promoting ageing [54,59]. These mechanisms typically include telomere shortening and the de-repression of the *INK4a* locus, which serve the purpose of preventing cellular proliferation, which in turn decreases cancer risk, but promotes ageing [59].

In addition to the molecular changes in cancer and ageing, there are key common changes in both processes that affect the extracellular space. Specifically, the crosslinking of collagen with fibulin, fibrillin and elastin improves the structural stabilisation of the ECM. In younger skin, collagen fibres intersect with each other at 90° angles, a pattern that breaks down with ageing, thereby contributing to mechanical and structural changes that cause the skin to wrinkle [59,60]. Kaur et al. [59] analysed young versus aged fibroblast secretomes and showed that ageing markedly decreased the secretion of proteins, such as fibulin, agrin and, most importantly, hyaluronan and proteoglycan link proteins (HAPLN1), which are involved in the production and remodelling of the ECM. The loss of function of HAPLN1 during ageing has a significant effect on tumour cell motility [59]. Moreover, during ageing, there is also a decrease in the immune response function of T cells to infections, injuries, and cancer. However, previous studies on immunotherapy found that, in patients under the age of 50, the ratio of clusters of differentiation 8 (CD8) to regulatory T cells (CD8: T_reg_) decreased, making the patient less responsive to anti-programmed cell death protein-1 (PD1) than older patients [61]. When tested on young mice, depleting T_reg_ increases sensitivity to anti-PD1 therapy. However, although *HAPLN1* does not lower the CD8: T_reg_ ratio, HAPLN1 may alter the capability of neutrophils and polymorphonuclear myeloid-derived suppressor cells (PMN-MDSCs) to reach the tumour site [59].

There are several other changes occurring during ageing, which cannot be reversed merely by the addition of a single protein, such as HAPLN1. However, ECM changes by age suggest that they will affect immunotherapy responses, as they alter the ratio and proportion of immune cells in the tumour microenvironment. Because immunotherapy has had remarkable success in melanoma, improving the immune microenvironment through the promotion of a favourable CD8: T_reg_ ratio could lead to better responses [59]. Moreover, these studies show that by understanding the structure, composition, and properties of the tumour microenvironment during ageing, better skin models can be developed to improve the effectiveness of current therapies.

### 2.3. Clinical Management

Cancer diagnosis and therapy have significantly improved in the last decades. However, the mortality rate remains high with over 17 million diagnoses worldwide each year out of which over 9.6 million are terminal [62]. In the UK, Melanoma is the fifth most common cancer with 16,700 people being diagnosed every year out of which 2300 are fatal outcomes [63]. Early diagnosis of melanoma leads to a higher survival rate (5-year survival is 99%), whilst the later the diagnosis and more advanced the cancer is, the lower the survival rate (5-year survival ~15%) [64]

Currently, there are several types of therapeutic treatments available for patients diagnosed with melanoma. The standard treatments used for the removal of tumours or alleviating symptoms include surgery, immunotherapy, targeted therapy, and less frequently chemotherapy. Other forms of therapy, such as oncolytic virus therapy are also being researched for the treatment of melanoma.

- Surgery is conducted to remove the tumour and is the standard primary treatment for patients in stages I-IIIB. Excision includes safety margins of 0.5 cm for in situ melanomas, 1 cm for tumours with a thickness of up to 2 mm, and 2 cm for tumours thicker than 2 mm [8]. Depending on the location, the surgical defect may require skin grafting. Patients with melanomas that penetrate the dermis are offered a surgical intervention to test whether the melanoma has spread to the regional lymph nodes. If the cancer has spread, the regional nodes are removed with a lymphadenectomy. To increase the rate of survival, recent efforts are developing new adjuvant therapies, specifically immunotherapy, to prevent disease progression. New trials show adjuvant immunotherapy following primary tumour removal decreases disease progression.
- Immunotherapy/biological therapy is a type of treatment that activates the immune system to amplify the immune response against cancer. In this case, the following strategies are used [65,66]:
  - Immune checkpoint inhibitor therapy targets immune checkpoints (proteins found on T cells and some cancer cells), which are regulators of the immune system. Checkpoints help keep immune responses from being too strong, and cancer cells can activate them in order to decrease anticancer immunity. Targeting these checkpoints releases immunity, reinstating cancer-fighting immunity. Specifically, checkpoint inhibitors are used to restore the ability of T cells to destroy cancer cells. CTLA-4, a checkpoint found on the surface of T cells, is a common target for inhibition. When attached to protein B7 on a cancer cell, CTLA-4 prevents T cells from killing cancer cells, deactivating the T cells [53]. PD-1 and PDL-1 inhibitors are also used to target the PD-1/PDL-1 interaction, which occurs between T cells and cancer cells, respectively, inhibiting T cell activity. Some of these drugs include Ipilimumab, which targets CTLA4, Pembrolizumab and nivolumab, which are PD-1 inhibitors, and Atezolizumab, which is an anti-PDL-1 [65,67]. Immune checkpoint inhibitor treatment has revolutionised melanoma care for advanced stages, in monotherapy and in combination, and is showing promising results in the adjuvant setting. There are ongoing drug development efforts at the preclinical and clinical stages investigating new targets and strategies to awaken host immunity against cancer.
- Targeted therapy uses small molecules and immunotoxins to block the growth of cancer cells by interfering with specific targeted molecules known as “molecular targets” that are involved in the growth, progression, and spread of cancer [53,68]. Despite being from the same tumour, cancer cells can be highly heterogenous. Some common drugs used in targeted therapy for melanoma are vemurafenib, dabrafenib and encorafenib, which directly attack oncogenic BRAF and the MAP Kinase pathway. In contrast to cytotoxic agents, targeted therapies do not cause as significant side effects to normal non-cancerous tissues as the drugs act directly on specific molecular cancer targets [69].
- There are multiple new avenues of research in targeted and immunotherapy. One promising strategy is the development of oncolytic virus therapy, which uses a genetically engineered version of a naturally occurring virus, injected directly into the tumours in the skin and lymph nodes, to infect and break down cancer cells without harming healthy cells. Talimogene Laherparepvec (T-vec), a modified herpes simplex virus, is a common genetically engineered oncolytic virus, able not only to suppress the growth of tumours but also to prolong overall survival [70].

## 3. 3D in Vitro Models for Melanoma Modelling

Much of our current body of knowledge on melanoma is based on the use of 2D monolayer cell culture models and experiments usually conducted under ideal conditions not able to mimic the physiology of actual tissues, which can lead to the over- or underestimation of the effectivity and dosage of a drug [71]. Nevertheless, in vitro 2D melanoma cell culture models are easy to establish and afford high-throughput screening. However, they do not accurately recreate either the native tumour microenvironment or the pathophysiological conditions and are a simplistic representation compared to 3D and in vivo models.

Alternatively, animal models are a powerful tool in understanding melanoma. An extensively used preclinical model is the murine xenograft which involves the grafting of established or primary (including patient-derived) human melanoma cells or tumour tissue into immunodeficient mice [72]. However, murine models do not accurately replicate human skin physiology or the melanoma microenvironment at both the primary and metastasised site. Furthermore, the high cost of animal maintenance coupled with the ethical and regulatory drive towards the reduction, refinement, and replacement (3Rs) of animals in studies requires new models to be developed to complement or replace an in vivo study [73].

The development of more effective anti-cancer drugs and therapies requires a deeper understanding of the interactions between cancer cells, neighbouring healthy cells, the ECM, and surrounding tissue and components, which unfortunately are not yet reproduced by existing models [74]. To study the complex cell–cell and cell–ECM interactions occurring in the TME, sophisticated biomimetic platforms are required to enable investigation of key features of cancer cells and their behaviour, such as migration and proliferation [75]. Such models have proven to be difficult to produce as the in vitro and in vivo models fail to mimic and reproduce the exact complex tumoural and non-tumoural interactions [75]. Therefore, there is a significant demand for improved models able to capture the complexities and heterogeneities of cancer biology.

Significant progress in creating improved melanoma models has been made [76]. A repertoire of 3D in vitro models, such as spheroids [77] organoids [78,79,80], microfluidic platforms [81], human skin equivalents (HSEs) [50,82,83,84,85,86,87,88,89,90,91,92], and bioprinting [1,83,86,93,94,95,96,97,98,99,100,101,102,103,104,105,106,107,108] have been developed or have the potential to be used for the investigation of melanoma. Combinatorial strategies based on the principles of tissue engineering and bioprinting technologies are emerging as a viable route to creating biomimetic platforms for disease modelling. Such models aim to allow a better understanding of the physiology of the disease, which is essential to creating patient specific models, and developing effective drug testing platforms.

### 3.1. Multicellular Tumour Spheroids and Organoids

**Figure 2:**
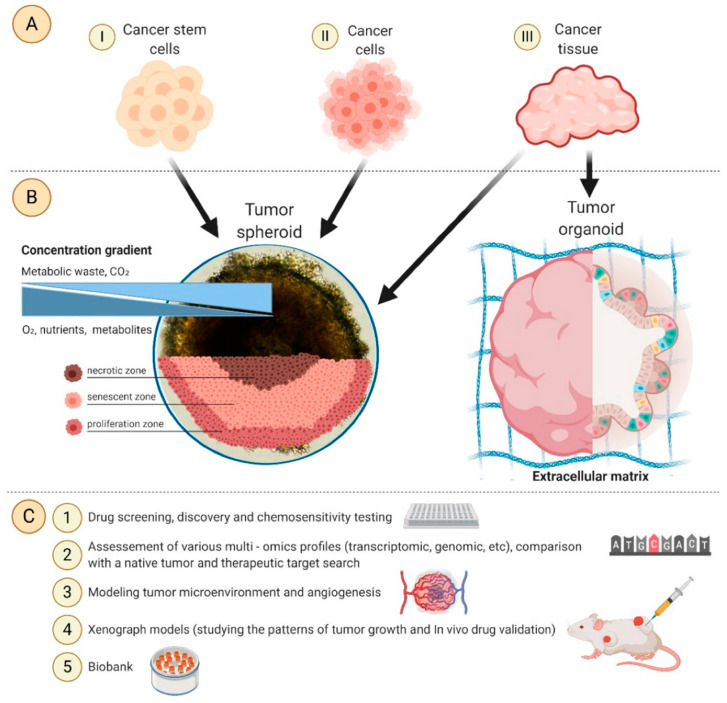
(**A**) Cells and tissues used to develop spheroids and organoids. (**B**) Structure, concentration gradients, and cell state in the spheroid. (**C**) Application areas. Image from reference [78].

Cell spheroids or multicellular tumour spheroids (MTCS) are 3D structures that are fabricated by a process of cell aggregation and self-assembly via cell–cell adhesion (Figure 2) [78,109,110]. Spheroids are gaining significant interest as they simulate the growth of the tumour in vivo and the corresponding microenvironment better than 2D models. MTCS exhibit key characteristics, such as cell–cell adhesion; hypoxic core surrounded by proliferating cells; limited drug penetration, thus closely mimicking real drug-tumour interactions and a gradient in oxygen, metabolites, and nutrients, making them highly relevant for melanoma studies [76,78]. Melanoma tumour spheroids are usually formed by the co-culture of melanoma cells with other cell types, such as fibroblasts and keratinocytes, providing useful platforms for drug screening [111].

Spheroids can be manufactured through several techniques, ranging in complexity, including hanging drop, low attachment plates, rotating flasks, magnetic levitation, microwell scaffolds, and microfluidics (Table 3) [112]. Spheroid size heterogeneity, throughput, and reproducibility are key challenges in the fabrication process.

The CD271 neurotrophin receptor is a member of the tumour necrosis factor receptor superfamily which regulates apoptosis in many cell settings [76]. It has been shown that CD271 is significantly expressed in early-stage melanomas and it is progressively lost when the tumour progresses which shows an inverse correlation with hypoxia inducible factor (HF-1α) [128]. Saltari et al. [129] investigated the role of CD271 in melanoma using spheroids to mimic the heterogeneity of melanoma. Saltari et al. [129] showed that CD271 is inversely correlated to the invasiveness and growth of the tumour. Results showed that *CD271* decreases when the tumour progressed from primary to metastatic melanoma in the same patient. When implanted into a collagen I matrix, CD271 was highly expressed in those cells which were confined to the body of the sphere. However, CD271 was almost absent in the cells that were detached from the spheroid [129]. These results contradicted previous observations that showed that neutrophins in fact regulated the progression of melanoma [130]. This conflicting observation was also confirmed in a study conducted by Saleh et al. [131], which showed a higher percentage of melanoma CD271 in 2D cell culture models and can be explained by the different types of models used as early studies were conducted using 2D models.

Spheroids have been shown to maintain several additional melanoma specific markers, such as HIF-1α, ABCB5, and Oct4 which were freshly isolated and derived from the same patients for up to 168 h [129,132,133]. A study by Koroknai et al. [134] provided data on gene expression differences between traditional 2D and 3D melanoma cell culture, as well as detailed analysis to clearly show gene expression differences between *BRAF* inhibitor (*BRAFi*) sensitive- and resistant melanoma spheroids. The data presented, clearly demonstrate the significant differences in gene expression between traditional and 3D cell culture, and these findings may be useful in better understanding the resistance profile of melanoma cells in tumour tissue [134].

Budden et al. [135] performed a study to determine if the damage caused by UVR degradation could affect melanoma progression in spheroids that contain increasing collagen concentrations. It was found that three cell lines exhibited different UVR mutation signatures. Although the UVR history of melanoma cell lines did not prevent the invasion of three melanoma lines, the concentrations of collagen were optimal for their invasion in 1.5 mg/mL collagen, and higher (2.5 mg/mL, *p*  <  0.0001) and lower collagen concentrations significantly reduced invasion into the ECM (0.25 mg/mL, *p*  <  0.0001, 0.5 mg/mL, *p*  =  0.03. Additionally, it was shown that melanoma cells can invade the spheroid without causing any damage to the surrounding tissue. They were also able to generate organotypic dermal constructs with high and low collagen densities. UV human foreskin fibroblast cell line (UV-HFF) constructs exhibited decreased fibronectin and elastin expression. These data support the notion that melanoma invasion is optimal in terms of collagen concentrations [135].

**Figure 3:**
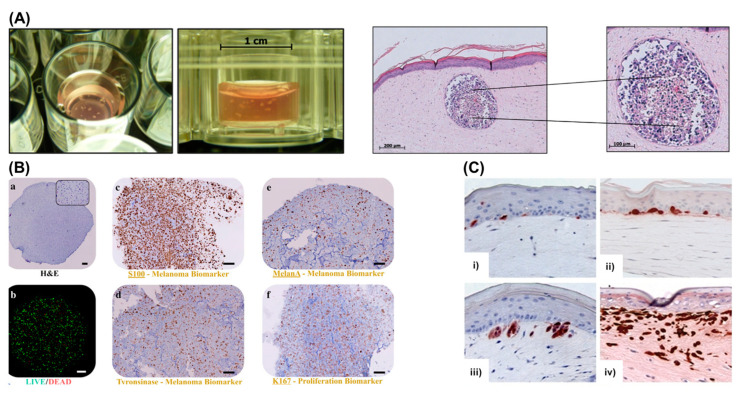
Examples of melanoma models. (**A**) Melanoma spheroids (white spots) embedded within the dermal region of a 3D organotypic human full skin model (**left**); H&E staining showing sections of tumour spheroids containing a peripheral proliferating subpopulation and a central subpopulation mainly consisting of shrunken, apoptotic or necrotic cells (**right**). Reprinted with permission from Ref. [77]. Copyright 2018, JoVE; (**B**) Melanoma organoids: (**a**) H&E staining showing cell distribution; (**b**) LIVE/DEAD staining at day 7 (green: calcein AM-stained viable cells; red: ethidium homodimer-stained dead cell nuclei); immunohistochemistry for specific biomarkers: (**c**) S100 beta, (**d**) tyrosinase, (**e**) melanA, and (**f**) KI67. Reprinted with permission from Ref. [80]. Copyright 2019, Spring; (**C**) Three-dimensional skin models of different stages of melanoma: (**i**) normal melanocytes located at the basement membrane; (**ii**) RGP melanoma WM35 cells growing as cell clusters in the epidermal layer; (**iii**). VGP melanoma WM793 cells invading into the dermis via the basement membrane; (**iv**) metastatic melanoma 1205Lu cells aggressively invading the dermis. Reprinted with permission from Ref. [144]. Copyright 2022, JoVE.

In another approach, melanoma spheroids were also integrated into a 3D organotypic human full skin model for modelling the human cutaneous melanoma metastasis (Figure 3A) [77]. A similar approach was also explored to engineer a 3D melanoma model containing blood and lymphatic capillaries, which was responsive to the treatment with a selective inhibitor of *BRAF* (vemurafenib) in a dose-dependent manner [136].

Organoids are complex 3D culture systems that recapitulate key the structural and functional characteristics of an organ. The establishment of organoids relies on cell differentiation and self-organisation of either tissue-resident adult stem cells obtained from biopsy samples or pluripotent stem cells (PSCs) [137]. Melanoma organoids have been created from patient tissue specimens (stage III or IV melanoma) for personalised immunotherapy screening. After processing, unsorted cell populations were suspended in a hyaluronic/collagen hydrogel precursor solution supporting the formation of organoids (Figure 3B) for subsequent drug screening [80].

### 3.2. Tumour-On-A-Chip

Organ-on-chip and tumour-on-a-chip models are being widely developed to reconstruct the complex microenvironment in vitro and to overcome some limitations in organ type cultures, such as inefficient tissue perfusion [76,138,139,140]. The microfluidic platform is a device where living cells can be continuously cultivated and infused in microscopic sized chambers, allowing the controlled release of growth factors or nutrients. Using this method, Ayuso et al. [81] co-cultured melanoma cells in the presence of dermal keratinocytes and fibroblasts. The results showed that the presence of dermal fibroblasts and keratinocytes caused changes to the morphology and growth pattern of melanoma cells. Molecular analysis revealed changes in the chemokine secretion pattern and identified a variety of secreted factors involved in tumour progression. Finally, the metabolic optical images showed that melanoma cells, fibroblasts, and keratinocytes exhibit different metabolic characteristics. Furthermore, the presence of stromal cells leads to the metabolic transformation of melanoma cells, highlighting the effect of the skin microenvironment on the evolution of melanoma [81].

### 3.3. Reconstructed 3D Skin Equivalents

For the development of effective anti-cancer drugs, it is vital to understand the interactions between cancer cells, neighbouring healthy cells, the ECM, and surrounding tissue and components [141]. Tissue engineering has been used to create HSE models, mimicking the human skin behaviour, to understand the mechanistic behaviour of tumours within an in vivo-like environment [142]. HSEs are advanced 3D models obtained from biopsies and created either manually or using bioprinting technology. These methods use reconstructed skin from isolated primary human cutaneous cells along with the ECM components (mainly collagen) [92]. After the formation of each skin layer, the model is exposed to an air–liquid interface allowing keratinocyte differentiation and stratification [143].

Reconstructed in vitro 3D skin models allow for a sophisticated recapitulation of key aspects of the native tissue, assuming a pivotal role as versatile platforms for the study of melanoma behaviour in human skin. These models are typically created as a top layer of stratified, differentiated keratinocytes, a middle layer of the basement membrane, and a bottom layer of fibroblasts embedded within collagen type I. Patient-derived melanoma cells can be incorporated into such 3D skin models, allowing the recapitulation of the crosstalk between melanoma cells, keratinocytes, and fibroblasts, including paracrine signalling, cell–matrix and cell–cell interactions. It has also been demonstrated that patient-derived melanoma cells cultured within bilayer 3D skin models (Figure 3C) recapitulate stage-specific properties of melanoma cells, such as morphology and invasiveness [144].

Tumour cells, such as melanoma cells, could be incorporated into HSE, providing an excellent model for studying tumour growth and invasion as well as a platform for pharmacological analyses, saving time and money typically associated with animal experiments. In vitro generation of melanoma skin equivalent (MSE) has been described using a variety of methods. Initially, these protocols were created to generate healthy HSE, and melanoma cells were seeded with keratinocytes on the reconstructed dermis.

One method is to use human de-epidermised dermis (DED). DED uses a skin biopsy of a donor, which is then deprived of the epidermal layer by overnight incubation in sodium chloride and finally digested by Dispase II to remove the basement membrane [50,76]. Primary keratinocytes, fibroblasts and melanoma cells are seeded together on the DED and grown submerged for 24 h followed by 5–20 days of culture and air-liquid interface to allow stratification [50]. In this model, WM35 melanoma cells or SK-MEL-28 melanoma cells are used in addition to fibroblasts and primary keratinocytes [132]. The melanoma cells in this model proliferate, migrate, and invade the dermis as in the in vivo environment. However, the invasion of the primary cell line and metastatic cell line varies in the pattern of invasion with the SK-MEL-28 line invading faster than the WM53 cells. Since the skin is used from a human biopsy, the model offers a human skin microenvironment, but variability between donors can result in inconsistent melanoma migration [85].

Recently, a novel humanised HSE using Alvetex^®^ has been developed by Hill et al. [85]. Most skin equivalents, comprising a dermal layer generated from fibroblasts that are implanted into collagen matrices derived from bovine or murine sources, are not able to recapitulate the human skin microenvironment and the ECM components [85]. To solve these limitations, Hill et al. [85] developed a full thickness model (organotypic skin culture). The HSE is produced by incorporating human fibroblasts to generate the dermis. The advantage of this system is that it overcomes the limitations of the previous model which uses collagen derived from bovine or rat tails and does not replicate the human skin microenvironment. This scaffold allows the growth of fibroblasts, which are stimulated to produce their own ECM constituents, and which form a stable human-like dermal compartment [76]. Additionally, in this method, melanoma cells are seeded onto the dermal equivalent prior to the seeding of keratinocytes which places them in their natural microenvironment [85].

## 4. Bioprinting Skin and Melanoma Models

The interest in using bioprinting to fabricate artificial skin has significantly increased in the last decade. This has been mainly motivated by the unprecedented ability of these technologies in depositing cells, biomaterials, and bioactive molecules in predefined 3D locations with high precision and reproducibility [145,146,147]. Bioprinted HSEs, exhibiting varying levels of biological function and complexity at structural, material, and cellular level, has been fabricated using different bioprinting technologies including vat polymerisation [148,149], inkjet [87,108,150,151], laser-assisted [152,153], and extrusion [84,87,88,97,107,116,154]. In vitro models of skin have experienced important advances as demonstrated by the bioprinting of multilayer tissue constructs comprising multiple cells, such as fibroblasts, endothelial cells, adipocytes, pericytes, stem cells, induced pluripotent stem cells (iPSCs), melanocytes, and keratinocytes [1,86,93,97,105,108].

A major advantage of bioprinting relies on the ability to closely mimic the architecture of the native skin through the deposition of bioinks comprised of skin cells and bioinstructive materials into a 3D construct with a tissue-specific organisation. It has been demonstrated that increasing the complexity and biomimicry of bioprinted skin translates to improved biological function, predictive value, and healing ability [1,116]. The feasibility of skin bioprinting has been demonstrated not only in vitro but also in situ by pre-clinical studies showing that the deposition of cells (stem cells, fibroblasts and/or keratinocytes) directly onto the wound bed of mouse or porcine models stimulates healing [94,155]. Although bioprinted in vitro skin has primarily been used as grafts for skin repair, a great interest is now focused on creating advanced tissue models capable of modelling key biophysical, biochemical, and biological features of both healthy and diseased skin. Such models are appealing and urgently needed as platforms for the screening and validation of drugs and cosmetics, as well as to perform fundamental studies to unravel mechanisms underlying skin diseases. Efforts have also been made in creating 3D bioprinted models of skin diseases, such as atopic dermatitis [88], psoriasis [156], and cutaneous melanoma [157,158]. However, the reported achievements in bioprinting diseased skin models are more modest so far. The following sections provide an overview of the design considerations for bioinks, bioprinting technologies, and advancements in bioprinted skin models to provide the contextual background and understanding required to develop a bioprinted melanoma model. Such topics are reviewed in detail here [83,106,146,147,159,160,161,162,163,164,165,166,167,168,169,170,171,172,173].

### 4.1. Bioinks

The effective fabrication of a multicellular and structurally complex bioprinted HSE requires the use of a suitable bioink. A bioink is a cell formulation that can be processed using biofabrication technologies and that can optionally contain biomaterials (e.g., hydrogel precursors) and bioactive molecules (e.g., DNA and growth factors) [162]. In contrast, a biomaterial ink formulation has no cells and only encounters cells after fabrication, for example, through cell seeding. Both approaches, and their combination, have been explored in skin bioprinting.

**Figure 4:**
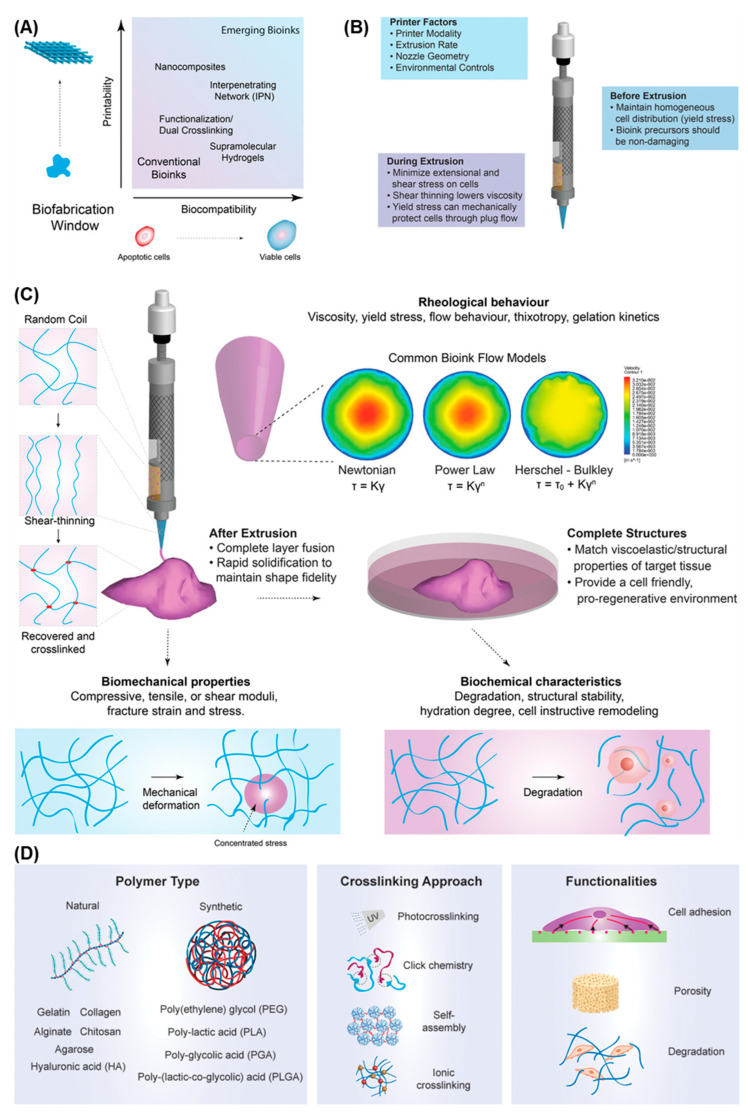
Factors in bioink design and development. (**A**) Bioinks require a balance between biocompatibility and printability, the biofabrication window, to enable them to be suitable for tissue engineering applications. Advancements in multicomponent bioink design are easing the fabrication process and improving biofunctionality. (**B**) Key printing factors to consider during extrusion-based printing of a bioink. (**C**) Rheological behaviour of the bioink influences printability, shape fidelity, and cell viability of the bioprinted construct. The biomechanical and biochemical properties of the bioink are crucial in determining the short and long-term cellular response. (**D**) The polymer type and crosslinking approach utilised are key in designing bioinks with specific biophysical and biochemical functionalities. Image adapted with permission from Refs. [159,161]. Copyright 2022, John Wiley and Sons.

A range of bioink design parameters must be considered during the development of a suitable bioink for skin bioprinting (Figure 4). Bioinks are typically composed of pre-crosslinked hydrogels or hydrogel precursors that are crosslinked during or post-fabrication into 3D hydrogel networks [161,163,164,169,174,175,176]. Hydrogels are a popular class of materials for bioink design due to their hydrophilic nature, which allows for the absorption and retention of large amounts of water, and resemblance to the hydrated state of native ECM. The biomaterial selection and crosslinking reaction are crucial in determining the bioink biocompatibility (e.g., cell viability, adhesion, proliferation, and differentiation), printability (e.g., rheology and shape fidelity), biomechanics (e.g., resemblance to target tissue), and biodegradation (e.g., match rate of tissue formation). For example, the selection of the optimal biomaterials for a bioink is a significant challenge. Natural biomaterials are typically biocompatible, biodegradable, versatile and can exhibit native cell binding and instructive motifs (animal derived biomaterials) [163,164,166,169,172]. However, despite excellent biological properties, natural materials often have poor mechanical properties, which commonly translates into limited printability. Alternatively, synthetic biomaterials display superior mechanical properties and stability, which can be explored to improve printability. However, synthetic materials lack native cell instructive motifs thus limiting biocompatibility and biodegradation [163,164,169,171,177,178].

Subsequently, a range of approaches to modify specific bioink attributes have been explored to improve printability and biocompatibility [159,160,167,168,174,175,179]. Hydrogel precursors can be modified through the addition of functional groups, for example, to allow chemical crosslinking (e.g., gelatin functionalised with methacryloyl) or the incorporation of cell instructive motifs (e.g., RGD) to promote cell attachment. A myriad of chemical (e.g., enzyme catalysed reactions, photopolymerisation, and click chemistry) and physical (e.g., thermal, ionic, and hydrogen bonding) crosslinking schemes are nowadays utilised in bioink design to tune printability and cell response of hydrogels. Novel dynamic and responsive bioinks that can broaden the biofabrication window and provide a more biomimetic environment for cells are being developed [159,160,167,168,174,175,179].

However, single material bioinks can struggle to meet the requirements of both printability and biocompatibility, hence the development of multicomponent bioinks [159,160,167,168,174,175,179]. These include interpenetrating networks, nanocomposites, supramolecular, and multi-material dual-crosslinked hydrogels which enable improvements in printability and biological functionality.

### 4.2. 3D Bioprinting Technologies

**Figure 5:**
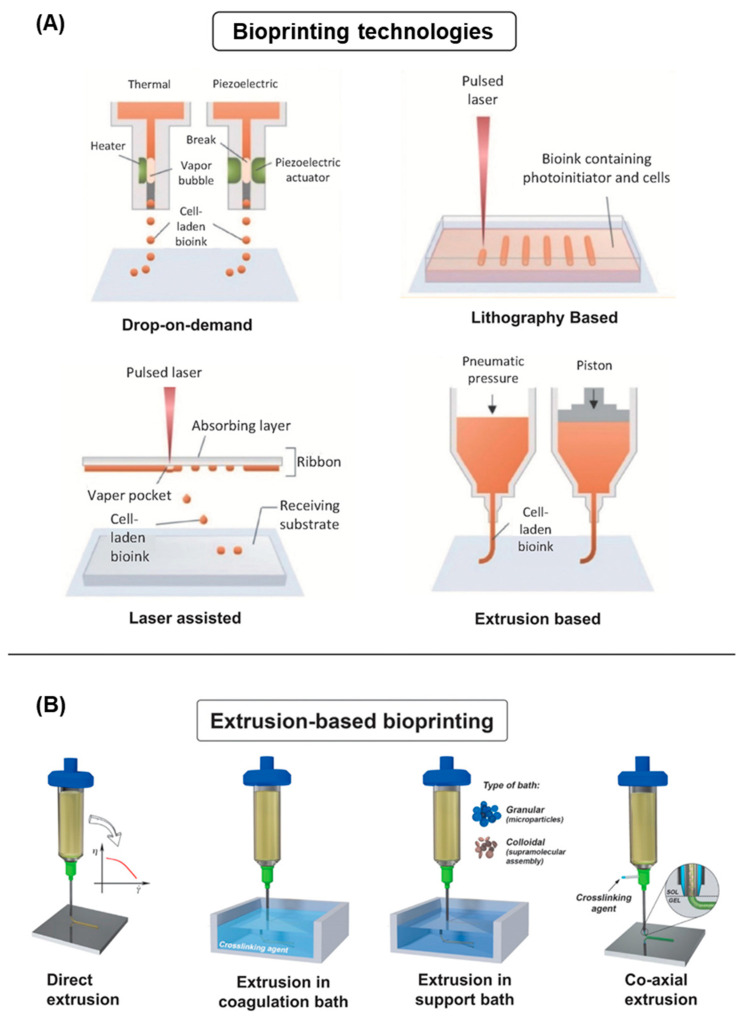
(**A**) Bioprinting technologies and (**B**) extrusion-based bioprinting strategies. Image adapted with permission from Ref. [160]. Copyright 2022, John Wiley and Sons.

Three-dimensional bioprinting encompasses a range of additive manufacturing technologies that can process bioinks into structurally complex and multi-material tissue constructs with high reproducibility. Bioprinting systems can comprise multiple printheads and technologies within a single machine working in tandem to allow multi-material printing. As the skin is a complex organ with a multicellular, multi-layered, and hierarchical organisation; the bioprinting technology selected should be able to replicate this architecture and be suitable for the specific bioink rheological properties. For skin bioprinting, the most relevant techniques are extrusion, material jetting (inkjet), laser assisted, and lithographic bioprinting (Figure 5A).

Extrusion-based bioprinting comprises systems that use pneumatic (compressed air), mechanical (screw/piston), or solenoid driven printheads to deposit bioinks onto a platform [106,160,161,165,168,173,180,181,182,183]. The majority of skin bioprinting studies utilise extrusion-based printing. Extrusion-based techniques are versatile, simple, low-cost, and widely used as a broad range of bioink viscosities with high cell densities can be processed into structures with high vertical shape fidelity. The bioinks should exhibit shear thinning or thixotropic properties. In comparison to other bioprinting techniques, extrusion presents lower printing resolution and cell viability (40–80%). The slow printing speed, especially with highly viscous materials, and the resulting shear stresses experienced by the cells in the nozzle (decreasing nozzle diameter increases shear stress) can compromise cell viability. Furthermore, extrusion bioprinting has some limitations in terms of precisely managing the volume of printed material during the lag period between switching the pressure on and off with the added potential risk of the nozzle clogging. The flexibility of extrusion-based bioprinting is also due to the variety of printing strategies that can be used, such as the use of coagulation baths, support baths, and co-axial extrusion (Figure 5B) [184,185,186]. These strategies can broaden the types of bioinks used, enabling difficult to print biomaterials, such as collagen to be accessible, and allowing unique construct and fibre architectures to be printed that are not possible through conventional extrusion.

Material jetting, continuous inkjet (CI) or drop on demand (DOD), is a bioprinting technique that deposits discrete bioink droplets in a layer-by-layer process onto a platform [106,164,168,173,187,188,189,190]. DOD uses an actuator (thermal, acoustic, piezoelectric, valves) to generate droplets of a specific size. Alternatively, CI exploits the Plateau–Raleigh instability of a flowing fluid to form discrete droplets. Material jetting is a low-cost technique with high print speed, resolution (20–100 µm), and cell viability (>85%). The bioinks used should present rheopectic properties to minimise shape change of the droplet after deposition. Although material jetting has a high print resolution the vertical fidelity of printed structures is low. The process is limited to printing low viscosity materials (<10 mPa s) with low cell concentrations (<10^6^ cells mL^−1^), with the additional problem of nozzle clogging at high viscosities. Furthermore, acoustic and thermal actuators can impact cell viability due to the frequencies and temperatures used.

Laser-assisted bioprinting (LAB) consists of three main elements: a pulsed laser source, a donor slide (ribbon), and a collector [164,168,191,192,193,194,195]. The donor slide comprises a laser transparent support (glass) with a thin coating of an energy absorbing metal layer (e.g., gold or titanium) and a final bioink layer. Laser irradiation and absorption of the energy at the focal point in the metal layer induces local evaporation and formation of an expanding bubble that causes a shockwave and jet formation that ejects a bioink droplet onto the collector. As LAB is a nozzle-free printing process, clogging is not a limitation. Furthermore, LAB is characterised by high printing resolution (droplets 10–100 µm) and cell viability (>95%), as well as the ability to print individual cells with high spatiotemporal control. However, these systems are expensive limiting their utilisation. Furthermore, the heat produced by the laser irradiation and evaporation process can compromise cell viability, although advancements, such as absorbing-film assisted laser induced forward motion (LIFT) and matrix-assisted pulsed laser evaporation direct write (MAPLE-DW) have mitigated this. The bioinks used have a limited range of viscosities (1–300 mPa·s) and cell densities; they must adhere to the donor slide, and the printing process only allows for one bioink to be used at a time.

Lithographic bioprinting processes, such as stereolithography (SLA), digital light processing (DLP), and two photon polymerisation (2PP) use specific wavelengths and intensities of light to selectively polymerise a bioresin into a 3D structure [164,168,196]. The bioresin can consist of photoinitiators, reactive monomers/macromers, additives (e.g., photo absorbers and radical scavengers), and cells. Lithographic techniques generally have high resolution with DLP achieving ~25–50 µm, whilst 2PP has a significantly higher resolution of ~100 nm; however, the 2PP resolution comes at the cost of low print speed and small maximum size of the construct. Cell viability is typically high (>85%) and moderate cell densities (<10^8^ cells mL^−1^) have been utilised. However, cytocompatibility issues arising from the choice of photoinitiator, wavelength, and light intensity must be considered. Visible light and compatible photoinitiators are becoming an attractive alternative to traditional UV bioprinting due to the lower associated cytotoxicity [196]. The printing systems can be low-cost, especially commercially available printers adapted for bioprinting, however, specialised systems, such as 2PP are considerably more expensive. A low viscosity bioresin must be used to allow the unreacted resin to move out of the 3D structure during the printing process. This can cause issues with cell sedimentation, especially in constructs with long print times. An obvious limitation is that biomaterials are limited to photopolymerisable polymers, thus biomaterials must be modified to allow compatibility.

A new approach in lithographic bioprinting termed volumetric printing (also referred to as tomographic volumetric printing or computed axial lithography) can overcome some of the limitations of conventional bioprinting techniques [197,198,199]. This technique uses a rotating volume of bioresin that is selectively photopolymerised by a dynamic projection of 2D light patterns. This enables the fabrication of complex 3D structures within seconds to minutes in a non-layer-by-layer process. Overcoming long print times and associated cell viability issues, furthermore, the process is not restricted by conventional 3D printing structural limitations, such as support structures and z-layer lines.

### 4.3. Skin Models

Three-dimensional bioprinting has allowed the development of complex biomimetic models that begin to recapitulate the structure and composition of native skin. This is important as an advanced melanoma skin model should include epidermal, dermal, and hypodermal layers (full-thickness); appendages (e.g., hair follicles and sweat glands); pigmentation (melanocytes), and a functioning vasculature. The incorporation of all these into a single disease model is a major challenge. However, the complexity of understanding the melanoma TME, interactions with surrounding tissues, and disease progression require a complex and tuneable model. Nevertheless, despite the challenges significant progress has been made in the bioprinting of advanced skin models, which can be translated to cutting-edge models of melanoma [145,146,147].

**Figure 6:**
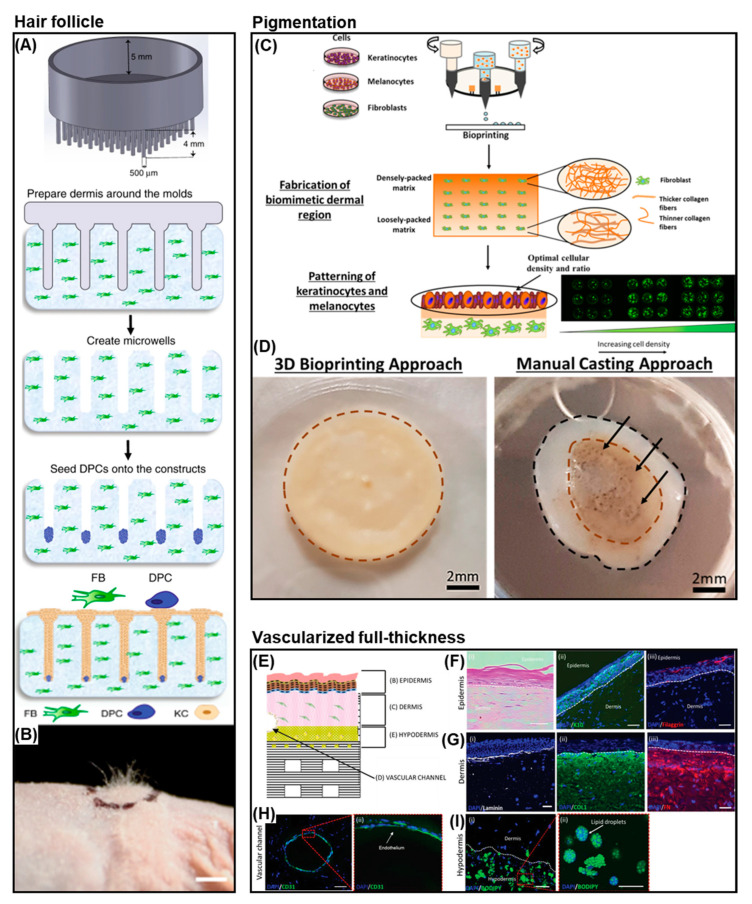
**Hair follicle:** (**A**) Schematic of the 3D printed mould and fabrication of microwells in a fibroblast encapsulated collagen hydrogel seeded with dermal papilla cells and keratinocytes. (**B**) Engraftment of the HSE in an immune-deficient nude mouse model showed hair growth after 4–6 weeks. Images from reference [200]. **Pigmentation:** (**C**) Bioprinting strategy for a pigmented biomimetic dermal and epidermal HSE containing melanocytes. (**D**) Uniform pigmentation is observed in the bioprinted skin construct and irregular pigmentation in the manually casted construct. Images adapted with permission from reference [108]. **Vascularised full-thickness:** (**E**) Schematic of the full-thickness HSE bioprinted structure including vascular channels and a hypodermis. Histological images of the (**F**) epidermis, (**G**) dermis, (**H**) vascular channels, and (**I**) hypodermis. Image reprinted with permission from Ref. [86]. Copyright 2022, John Wiley and Sons.

The bioprinting of skin appendages, such as hair follicles and sweat glands is an important direction in the development of biomimetic skin models due to their role in thermoregulation, homeostatic maintenance, and wound healing. Abaci et al. [200] developed a hair follicle skin model using 3D printing to create a high-resolution mould to pattern microwells inside a collagen hydrogel encapsulated with fibroblasts (Figure 6A). The microwells were seeded with dermal papilla cells, which spontaneously formed into aggregates, followed by the seeding of keratinocytes to create a hair follicle-like unit. In vitro results demonstrated differentiation of keratinocytes into specific hair lineages and prolonged culture even showed protrusion of hair follicles outside of the construct. In vivo engraftment of a vascularised construct, human umbilical vein endothelial cells encapsulated in the dermal layer showed human hair growth in a mouse model. Huang et al. [201] used extrusion-based bioprinting to develop a gelatin and alginate based bioink with epithelial progenitor cells, dermal homogenate, and epidermal growth factor. This aimed to recreate an inductive microenvironment to promote differentiation towards a sweat gland cell lineage. The results indicated that the bioink ECM-mimicking niche promoted sweat gland differentiation in vitro and in vivo was able to restore sweat gland functionality in a burn model. This approach was further developed to directly differentiate mesenchymal stem cells into a sweat gland cell [202] Additionally, Zhang et al. [203] refined the model further by combining the bioprinted sweat gland construct with the seeding of hair follicle spheroids to create a construct with both appendages.

Skin pigmentation is a major aspect of developing a viable bioprinted melanoma model. Therefore, studies on bioprinting melanocytes and the evaluation of melanin production are crucial. For example, Ng et al. [108] exhibited a two-step DOD bioprinting technique to fabricate 3D pigmented skin constructs using a collagen and polyvinylpyrrolidone bioink containing keratinocytes, melanocytes, and fibroblasts (Figure 6C). An initial dermal layer containing fibroblasts was printed and cultured prior to the precise deposition of melanocytes and keratinocytes droplets (1:8 ratio) and further cultured at the air–liquid interface. Histological analysis confirmed the presence of a mature stratified epidermis with uniform distribution of melanin. Similarly, Min et al. [105] printed a dermal structure consisting of multiple layers of collagen crosslinked with sodium bicarbonate and within these layers, a fibroblast bioink was embedded. Melanocytes were printed on top of the dermal layer either in a spot or full-layer pigmentation model followed by printing of keratinocytes and culturing at the air–liquid interface. The melanocytes showed dendritic formation in the epidermal layer and production and accumulation of melanin in a freckle-like morphology.

Vascularisation of skin constructs is important as the maximum gas and nutrient diffusion distance that can maintain cell survival is ~100–200 µm, hence, the long-term viability of the bioprinted skin construct relies on the creation of a functional vasculature [204,205]. Additionally, successful integration and viability of a HSE implant with the host tissue require either the implant is pre-vascularised and can rapidly integrate with the host tissue or is able to recruit and develop a vasculature via the host tissue. Furthermore, in melanoma a key mechanism for tumour growth and metastasis is via the vasculature, thus vascularised dermal and hypodermal components can provide a more in vivo-like environment that can promote cell–cell and cell–ECM interactions, vital for improved melanoma disease models. Using bioprinting, vascularised 3D HSEs can be engineered [86,149,206,207,208]. Yanez et al. [207] used inkjet bioprinting to fabricate a bilayer skin graft with human dermal microvascular endothelial cells embedded between a collagen dermal and epidermal layer containing fibroblasts and keratinocytes, respectively. The endothelial cell–thrombin bioink was printed into a fibrinogen layer to generate a fibrin vascular network. The in vivo study showed improved wound contraction, normal skin appearance, and the indication was that blood microvessels had formed and were integrating with the host tissue. An alternative approach by Abaci et al. [206] used 3D printing to create moulds to fabricate sacrificial alginate channels of specific vasculature patterns. Dermal and epidermal layers were cast on top of the vascular channels and the alginate was removed and the channels perfused with endothelial cells. The endothelial cells attached and coated the inner walls of the microchannels and exhibited appropriate barrier function. The vascularised HSEs promoted neovascularisation during wound healing in a mouse model.

As bioprinting technologies and bioink design have progressed, the ability to bioprint complex full-thickness HSE models has become possible [1,86,116,209,210,211]. These models include not only dermal and epidermal compartments, but additional aspects of skin, such as the hypodermis (trilayer), a vascular network, and appendages. This is a significant development in allowing the development of biologically relevant melanoma models. For example, Kim et al. [86] have developed a platform for the bioprinting of a full-thickness vascularised skin model that resembles native skin (Figure 6E). The platform based on extrusion and inkjet printing, the use of multiple bioinks, a printed transwell support, perfusable vascular channels, and vascularised dermal and hypodermal layers provide a complex microenvironment that can be further developed to include additional skin appendages or interrogated as a disease model. Furthermore, Jorgensen et al. [116] bioprinted a full-thickness trilayer HSE containing keratinocytes and melanocytes (epidermal layer); fibroblasts, microvascular endothelial cells, and follicle dermal papillary cells (dermal layer) and preadipocytes (hypodermal layer). An extrusion-based bioprinter was used to print a fibrinogen based bioink crosslinked with thrombin to form a fibrin hydrogel. The bioprinted constructs were cultured for four days prior to implantation in a full-thickness mouse wound model. The bioprinted HSEs accelerated wound closure through enhanced epithelialisation and produced a normal basket-weave collagen matrix, potentially minimising scarring. The HSE after implantation resembled phenotypically human skin and was a mixture of both the implant and infiltrating host cells.

### 4.4. Bioprinting in Vitro Models of Melanoma

Despite existing skin and melanoma models having been proved to be useful for understanding disease pathology, performing drug screening, and even providing scientific support to clinical trials, a major challenge remains in the development of a melanoma model capable of better reproducing the different features of the tumour microenvironment underlying the in vivo resistance and differential responses in human patients. This is an ambitious and complex task as melanoma cells are characterised by high genetic instability and plasticity, and the ability to dedifferentiate into a variety of states and secret factors that promote melanoma cell viability in an autocrine way and reprogram adjacent stroma cells that influence the tumour microenvironment in a paracrine manner [212].

**Figure 7:**
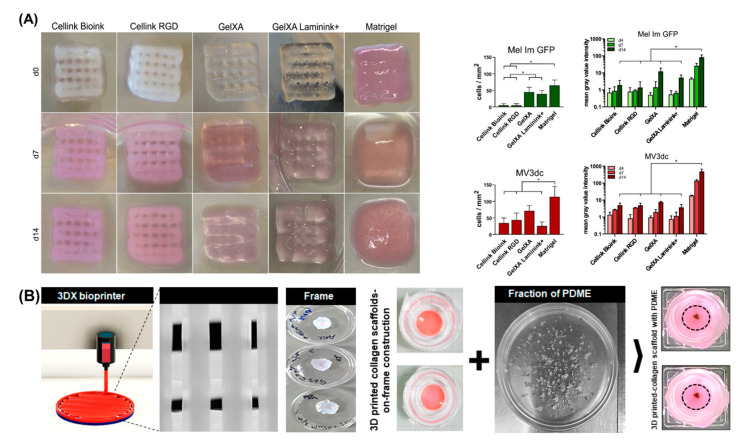
(**A**) Images showing the printing fidelity of bioprinted constructs (1 cm^2^) created with three layers and 105 melanoma cells/mL suspended within Cellink Bioink, Cellink RGD, GelXA, GelXA Laminink+ or Matrigel at different time points (**left**); influence of bioink on viable cells at day 1 (**middle**) and cell proliferation at day 14 (**right**). Images adapted from reference [157]. (**B**) Bioprinting of collagen scaffolds and loading of cryopreserved patient-derived melanoma explants to generate an in vitro 3D culture model of melanoma. Image from reference [158].

The potential of bioprinting to create biomimetic melanoma models has been demonstrated in a few studies focused on the bioprinting of melanoma cells and evaluation of cell response. In a recent study, the influence of bioink properties and composition on the proliferation and morphology of metastasis-derived melanoma cell lines (Mel Im and MV3dc) was investigated [157]. Extrusion-based bioprinting was used and results showed that the printing fidelity, cell viability and proliferation were highly dependent on the bioink material (Figure 7A). Although cells survived in bioprinted hydrogels, cell morphology, and proliferation were differentially affected by the tested bioinks owing to their distinct biophysical and biochemical properties. Despite the highest cell proliferation observed in Matrigel hydrogels, bioprinted constructs displayed poor shape fidelity and long-term stability, which limits the fabrication of 3D constructs with complex architectures. In a subsequent study, a more detailed characterisation was performed to correlate the properties (e.g., mechanical properties) of hydrogels (alginate, alginate dialdehyde crosslinked with gelatin, and thiol-modified hyaluronan crosslinked with polyethylene glycol diacrylate) with the response of melanoma cells, but using a manual approach to create the hydrogels instead of bioprinting [213]. Using a different strategy, extrusion-based bioprinting was explored to create collagen scaffolds to support the maintenance and survival of cryopreserved patient-derived melanoma explants seeded onto the bioprinted scaffolds (Figure 7B) [158]. The results showed improved cell maintenance and survival compared to standard 2D culture and retention of key melanoma biomarker expression.

**Figure 8:**
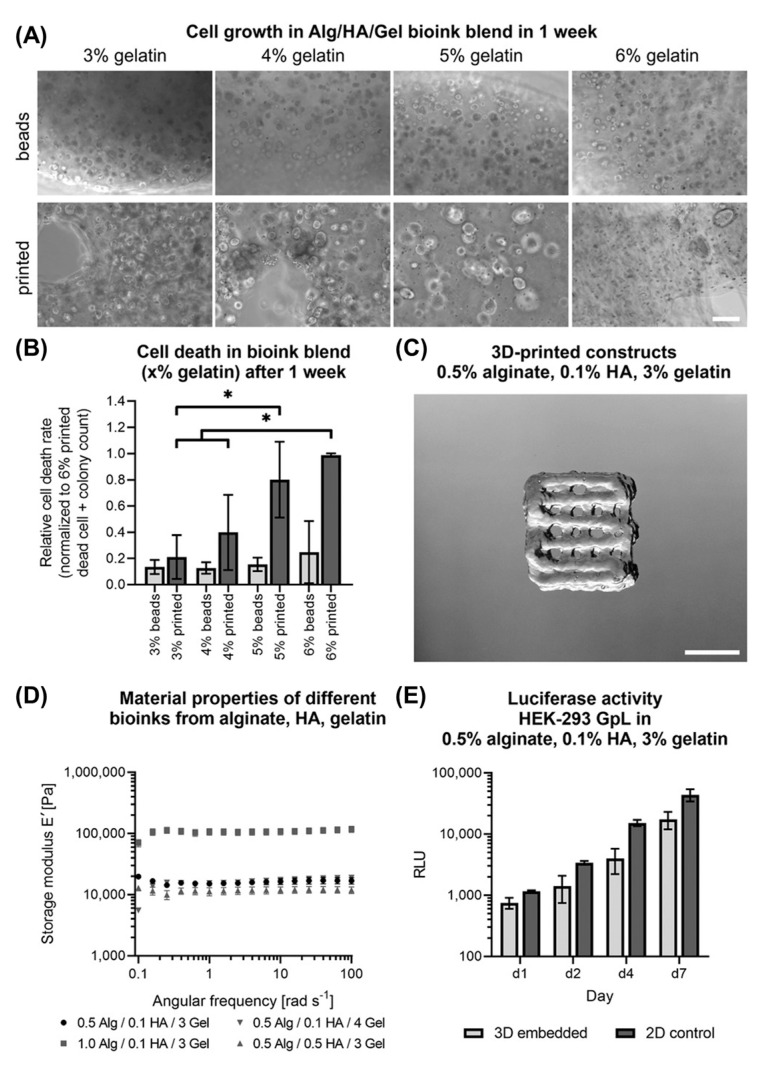
Alg/HA/Gel bioprinted melanoma scaffolds. (**A**) Melanoma cell growth in bioink after one week, (**B**) quantification of cell death in bioink with different gelatin concentrations, (**C**) printed construct of the bioink blend; (**D**) storage modulus of bioink blend, and (**E**) luciferase activity of supernatants of HEK-293 cells in 3D bioink blend and conventional 2D data. Image reprinted with permission from Ref. [214]. Copyright 2022, John Wiley and Sons.

Schmid et al. [214] developed a bioink consisting of gelatin, alginate, and hyaluronic acid (Alg/HA/Gel), which was successfully used for melanoma studies and mimics the tumour microenvironment (Figure 8). The results show that the developed bioink is suitable for extrusion-based bioprinting, providing good shape fidelity, and high cell survival post-printing. Moreover, in combination with an arteriovenous loop, the in vivo model provides a unique platform for studying melanoma progression, angiogenesis, and ultimately metastases similar to human pathophysiology, in an isolated and controlled environment. Furthermore, the bioink provides a highly defined material composition compared to other tumour models which rely on, for example, Matrigel which is a complex and poorly defined heterogenous basement membrane matrix derived from sarcoma cells that can have significant batch-to-batch differences in composition [215].

### 4.5. Challenges of Bioprinting Melanoma Models

Skin is a complex multi-functional tissue with anisotropic material, structural, and cellular properties. For example, the composition, density, and organisation of cells, appendages, and the ECM varies significantly between skin layers and within the different regions of the layers. Recreating this heterogeneity and architectural features, such as the wavy epidermal–dermal junction is a significant challenge in skin bioprinting.

This necessitates the development of enhanced software tools for designing and fabricating such complex constructs; advanced bioprinting systems for fabricating multi-material, hierarchical, and functionally graded skin constructs with high resolution and printing speed.

The selection of a bioink for a melanoma model will differ from a bioprinted skin graft. The specific requirement of each application means that the properties of the bioink will be distinct to provide specific microenvironmental cues to both normal and cancer cells. Skin grafts may favour long-term construct stability and promotion of host vasculature in-growth, but the bioink itself may not need to fully recapitulate the native ECM to fulfil its purpose of being a successful skin graft. Whilst a melanoma bioink should from the beginning as closely as possible resemble the native ECM to promote appropriate cell–ECM interactions and maintain markers of melanoma expression, which are crucial in understanding melanoma physiology and progression. Unmodified native skin ECM biomaterials, retaining all instructive and binding sequences, such as collagen may be the most desirable for this objective. Furthermore, with new bioprinting strategies, the difficulty of bioprinting collagen and other native polymers is no longer as much of a challenge [216,217,218].

The long-term viability of a bioprinted tissue requires the integration of a perfusable vascular network [205]. This is a key aim in tissue engineering to fabricate viable large-scale tissues and organs for implantation and integration with the host tissue. Additionally, the long-term study of biofabricated disease models is essential in developing a new understanding of pathophysiology and replacing equivalent animal models. This is especially important in cancer models due to the utilisation of the vasculature by tumour cells for angiogenesis, intravasation, metastasis, and circulation. Although vascularised melanoma skin models have not yet been developed, the tools and platforms are available and been demonstrated in bioprinted HSEs.

## 5. Conclusions and Future Perspectives

Malignant melanoma is an aggressive form of skin cancer that can be fatal. It is, therefore, essential to understand the genetic drivers and triggers underlying melanoma onset and progression, as well as the dynamics of the tumour microenvironment. While existing 2D models have provided us with core concepts of cancer progression, they are not fully viable for cancer studies as they are conceived under ideal and non-relevant physiological conditions. To overcome these issues, 3D models, such as spheroids and enabling technologies, for example 3D bioprinting have been used to create advanced models that closely recreate the microenvironmental conditions of native tissue as well as the cell–cell and cell–ECM interactions. As a result, a repertoire of melanoma models is available, comprising 2D and 3D tissue cultures, spheroids, organoids, and animal models, such as genetically engineered mice and patient-derived xenografts.

Bioprinting is assuming a central position in the biofabrication of 3D melanoma models with improved biomimicry. This has been made possible by translating the understanding gained through the steady development of advanced bioprinted HSE models. Indeed, by developing suitable bioinks and bioprinting strategies it is now possible to create 3D heterogeneous cancer models recapitulating key features of tumour microenvironment through bioprinting. In the context of melanoma, significant progress has recently been made regarding the design of bioinks for the bioprinting of 3D melanoma models containing tumour-associated cells. Efforts have also been made in promoting the vascularisation of melanoma models, though the progress in this field has been more modest. Furthermore, there is still a gap in full thickness skin models to study melanoma as skin is a complex tissue that differs according to race (pigmentation which affects the amount of UVR absorbed), location of the tissue on different parts of the body (amount of hair follicles, sweat glands, and thickness), and age (elasticity and ECM degradation). In addition, further investigation is also warranted regarding the integration of immune cells and other key cells of the tumour microenvironment to better reproduce the native cellular composition. Bioprinting can also provide a platform for personalised melanoma models utilising patient-derived cells to develop patient specific treatment regimes.

Furthermore, advancements in bioprinting also open opportunities for developing models and furthering our understanding of rare non-cutaneous melanoma subtypes, uveal and mucosal. For example, bioprinting of the cornea is rapidly developing and future studies may begin to include the uveal layer [219,220,221]. Knowledge of other non-melanoma skin cancers, such as Merkel cell carcinoma, basal cell carcinoma, squamous cell carcinoma, and sebaceous gland carcinoma can benefit as well from the progress in bioprinting of HSEs.

Overall, we can conclude that novel developments in improved melanoma models that capture fundamental cell–cell and cell–ECM interactions are required to perform fundamental studies towards identifying new therapeutic targets and perform screening of the efficacy and safety of new therapeutic modalities.

## Figures and Tables

**Table 1:** Gene driver mutations in various stages of melanoma progression.

Pathway Activated	Gene	Common Mutations	Subtype	Role during Progression	Refs.
MAPK	*BRAF*	V600E	Non-CSD	Initiation	[18,22,23]
	*BRAF*	V600K, K601E and G469A	CSD	Initiation	[18,24]
	*NRAS*	Q61R (cutaneous melanoma), Q61K	CSD	Initiation	[18,23,25]
	*NFI*	Deletion which leads to no neurofibromin	CSD	Initiation	[18,23,26]
Telomerase	*TERT*	Mutations in h*TERT* promoter leading to deregulation of cell cycle and immortalisation of cancer cells	CSD and non-CSD	Progression	[18,23,27]
Retinoblastoma protein	*CDKN2A*	Disables mutations occurring throughout the protein	CSD and non-CSD	Progression	[18,23,28]
Chromatin remodelling	*ARID1A, ARID1B* and/or *ARID2*	Disables mutations occurring throughout the protein			[18,23,29]
PI3K	*PTEN*	Disables mutations occurring throughout the protein + deletions	Non-CSD	Advanced Progression	[18,23,30]
p53	*TP53*	Disables mutations occurring throughout the protein	CSD	Advanced Progression	[18]

MAPK—Mitogen- activated protein kinases; *TERT*—Telomerase reverse transcriptase; *CDKN2A*—cyclin-dependent kinase inhibitor 2A; ARID—AT-rich interactive domain-containing protein; *PTEN*—phosphate and tensin homolog.

**Table 2:** Melanoma progression stages. Reprinted with permission from Ref. [53]. Copyright 2022, Terese Winslow.

TNM	Stage in Progression	Description
N/A	**Melanocytic naevi (moles)**	Typically, benign proliferation of melanocytes Formed due to BRAFV600E mutations Dark brownish in colour due to the pigment melanin
0	**Melanoma in situ** 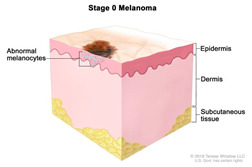	Proliferation of melanocytes with enlarged nuclei and irregular pattern growth within the epidermis Non-invasive precursor of melanoma [46] Contained in the initial epidermal area but have not progressed into deeper layers of the skin [46] Various genetic alterations developed over time [15].
I	**Invasive melanoma** 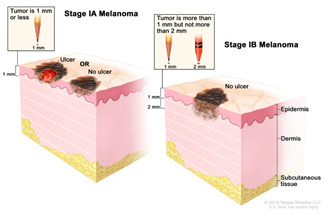	Tumour has grown with or without ulceration beyond the epidermis but has not spread to lymph nodes or metastasised Melanoma cells leave the epithelium of the epidermis and enter the mesenchymal tissue. Melanoma inherits driver mutations which activate the MAPK pathway along with the TERT mutations amassed during earlier stages. In the later stages of primary melanomas, late-stage mutations are observed, especially in the TP53 gene, the most vital tumour suppressor [47,48]. Key difference between TNM I and II is progression of melanoma into the dermis.
II	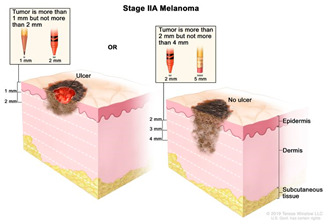	
III	**Advanced progression into lymph nodes**	Metastases to regional lymph nodes are a common indicator of cancer dissemination [48]. Mutations of the PTEN protein are also observed in the later stages [49].
IV	**Metastatic melanoma** 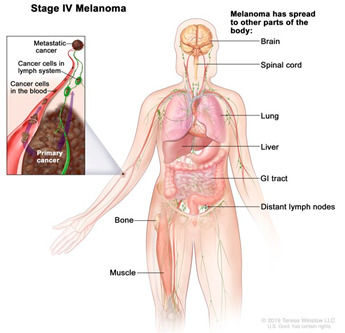	Metastasis occurs when cells from the primary site spread to a different secondary site and inhabit other tissues inside the host’s body

**Table 3:** Main methods to fabricate spheroids.

Method		Features
**Scaffold based** 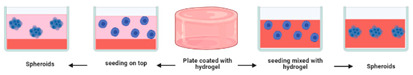	Porous non-adherent 3D scaffold which physically supports cell aggregation allowing formation of spheroids with a controlled size [113]	Advantages: good tensile strength compared to other methods [10] Limitations: Simplified architecture. Can be variable across lots [114]
**Hanging drop** 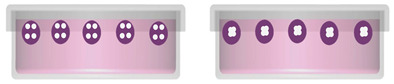	Drops of cell suspension are placed on the underside of a petri dish lid which hang due to surface tension. The cells then accumulate at the tip of the drop at air–liquid interface upon which they aggregate and form spheroids [115,116].	Advantages: Can produce ~384 spheroids per trial; controllability of spheroid size; does not require specialised equipment [10,117] Limitations: Risk of droplet dehydration; time required for spheroid formation; difficult to scale up [118,119]
**Magnetic levitation** 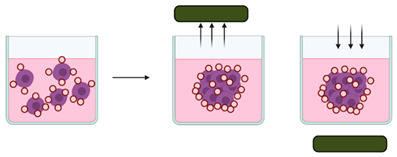	Cells are magnetised through a mixture of magnetic particles and incubated under magnetic forces to overcome gravitational forces, encouraging levitation and the formation of cellular aggregates [110,120].	Advantages: The speed of spheroid growth is high; forms intrinsic ECM; does not require specific medium [117] Limitations: Requires magnetic beads which can be expensive and toxic to cells; produces a limited number of spheroids [119,121].
**Spontaneous formation** 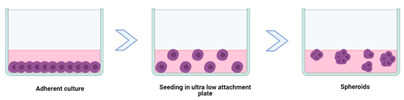	Uses ultra-low attachment plates coated with an inert substance (usually agar or poly-2-hydroxyethyl methacrylate (poly-HEMA)) which inhibits cells from attaching to the surface of the wells, thereby forcing cells to amass and form spheroids.	Advantages: Easy to use; inexpensive; large scale production [10]. Limitations: Low control over the size of the spheroids; spheroids are produced through a small number of cells, therefore, setting up the ratio of two different cell types in co-cultures can be difficult [10].
**Microfluidic platforms** 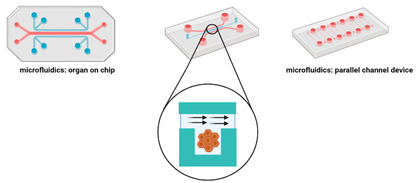	Cells are placed in microchannels with a free perfusion system which allows the continuous and uniform distribution of oxygen and nutrients and the elimination of waste [117]. This system can replicate the in vivo tumour microvasculature and guarantees the permeability of gases.	Advantages: Can mimic the tumour vasculature; high throughput drug screening; continuous release of oxygen and nutrients [122,123,124,125]. Limitations: Requires specialised equipment; post culture recovery can be difficult; difficult to precisely control the flow speed [122,123,124,125]
**Matrix encapsulation** ** 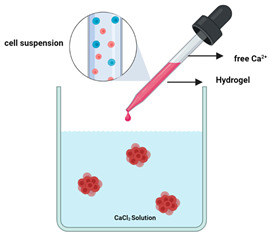 **	Suspended cells are surrounded by the hydrogel and placed in a calcium free solution which forms microcapsules (100 and 500 µm) in which cells aggregate to form matrix encapsulated spheroids [118].	Advantages: Enables cell–cell and cell–ECM interaction; simple to use and inexpensive [10,118] Limitations: High chance of cell necrosis due to confinement; size heterogeneity [10,118]
**Spinner and rotating flasks** 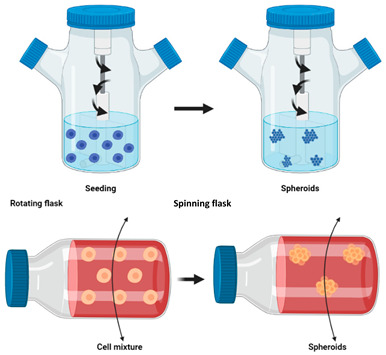	The medium is continuously agitated, inhibiting cell adhesion to the surface and leading to spheroid formation [117]. A magnetic stirrer is placed inside the spinner flask allowing the homogenous distribution of oxygen and nutrients. In continuous rotating flasks, the flask itself is rotated to allow the distribution of oxygen and nutrients.	Advantages: Large scale spheroid production; simple media changing; constant agitation provides continuous nutrients and oxygen transportation [10,118] Limitations: Cells undergo shearing under high agitation which can damage cells; slow agitation results in cell dispersion; spheroids are heterogenous ([10,118,121])
**Microcarrier beads** ** 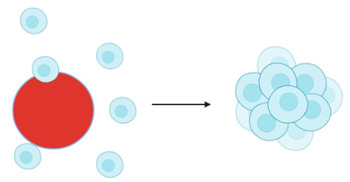 **	Cells adhere to the natural or synthetic matrix coated beads which form spheroidal structures [126]	Advantages: Fast and inexpensive method; produced spheroids are homogenous [88,126,127]. Provides a good cell attachment surface which allows aggregation, especially for those cells which are unable to aggregate spontaneously [117].
